# Immunological link between periodontitis and type 2 diabetes deciphered by single‐cell RNA analysis

**DOI:** 10.1002/ctm2.1503

**Published:** 2023-12-11

**Authors:** Hansong Lee, Ji‐Young Joo, Jae‐Min Song, Hyun‐Joo Kim, Yun Hak Kim, Hae Ryoun Park

**Affiliations:** ^1^ Medical Research Institute Pusan National University Yangsan Republic of Korea; ^2^ Department of Periodontology School of Dentistry, Pusan National University Yangsan Republic of Korea; ^3^ Department of Oral and Maxillofacial Surgery School of Dentistry, Pusan National University Yangsan Republic of Korea; ^4^ Department of Periodontology Dental and Life Science Institute, School of Dentistry, Pusan National University Yangsan Republic of Korea; ^5^ Department of Periodontology and Dental Research Institute Pusan National University Dental Hospital Yangsan Republic of Korea; ^6^ Periodontal Disease Signaling Network Research Center School of Dentistry, Pusan National University Yangsan Republic of Korea; ^7^ Department of Biomedical Informatics, School of Medicine Pusan National University Yangsan Republic of Korea; ^8^ Department of Anatomy School of Medicine, Pusan National University Yangsan Republic of Korea; ^9^ Department of Oral Pathology Dental and Life Science Institute, Pusan National University Yangsan Republic of Korea

**Keywords:** chronic inflammation, insulin resistance, periodontitis, single‐cell RNA sequencing, type 2 diabetes mellitus

## Abstract

**Background:**

Type 2 diabetes mellitus (DM) is a complex metabolic disorder that causes various complications, including periodontitis (PD). Although a bidirectional relationship has been reported between DM and PD, their immunological relationship remains poorly understood. Therefore, this study aimed to compare the immune response in patients with PD alone and in those with both PD and DM (PDDM) to expand our knowledge of the complicated connection between PD and DM.

**Methods:**

Peripheral blood mononuclear cells were collected from 11 healthy controls, 10 patients with PD without DM, and six patients with PDDM, followed by analysis using single‐cell RNA sequencing. The differences among groups were then compared based on intracellular and intercellular perspectives.

**Results:**

Compared to the healthy state, classical monocytes exhibited the highest degree of transcriptional change, with elevated levels of pro‐inflammatory cytokines in both PD and PDDM. DM diminished the effector function of CD8+ T and natural killer (NK) cells as well as completely modified the differentiation direction of these cells. Interestingly, a prominent pathway, RESISTIN, which is known to increase insulin resistance and susceptibility to diabetes, was found to be activated under both PD and PDDM conditions. In particular, *CAP1*+ classical monocytes from patients with PD and PDDM showed elevated nuclear factor kappa B‐inducing kinase activity.

**Conclusions:**

Overall, this study elucidates how the presence of DM contributes to the deterioration of T/NK cell immunity and the immunological basis connecting PD to DM.

## Graphical abstract

Myeloid cells establish a pro‐inflammatory milieu under periodontitis conditions, both with and without type 2 diabetes mellitus.

Type 2 diabetes mellitus impairs the effector function and differentiation of T/NK cells.

The RESISTIN pathway is a risk factor that makes periodontitis a precursor to type 2 diabetes mellitus.

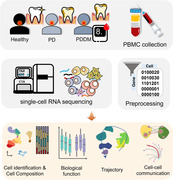

## 1 BACKGROUND

Type 2 diabetes mellitus (DM) is a multifaceted metabolic disease that alters glucose levels in the body. The global burden of DM has continuously increased since 1980, and according to the International Diabetes Federation, one in 10 adults had DM in 2021.^1^ DM is characterised by hyperglycaemia, which leads to low and chronic systemic inflammation.^2^ Patients with DM are well known to have elevated levels of inflammatory mediators such as interleukin‐1 (IL‐1), IL‐6, tumor necrosis factor α (TNF‐α) and monocyte chemoattractant proteins.^3^, ^4^, ^5^ This can result in long‐term impairment of many organs, such as the eyes, kidneys, nerves, heart and blood vessels, thus increasing susceptibility to other diseases.^6^

One of the major complications of diabetes is periodontitis (PD), which features destruction of the dental structure as well as low‐grade persistent systemic inflammation.^7^, ^8^ A Global Burden of Disease study reported that PD has a considerable impact worldwide, with a prevalence of 50%.^9^ Notably, PD is not merely a complication of DM. Although diabetes increases the risk of PD, periodontal inflammation is also known to complicate glycaemic control.^4^ Individuals with PD and DM have been reported to experience a reduction in haemoglobin A1c (HbA1c) levels following non‐surgical periodontal treatment.^10^, ^11^

Although the mechanism underlying the effect of PD on DM is unclear, low‐grade systemic inflammation is a possible risk factor contributing to diabetes.^12^, ^13^, ^14^, ^15^ Once bacteremia and endotoxemia occur in the dental system, locally secreted pro‐inflammatory mediators move into the circulatory system, leading to an increase in serum levels.^16^, ^17^ Moreover, persistent systemic inflammation can trigger activation of trained haematopoietic stem and progenitor cells. This results in hyperactivity of myeloid cells, eventually leading to organ impairment.^18^

Transcriptome analysis has been used as one of the approaches to uncover the immunological connections between PD and DM.^19^, ^20^, ^21^, ^22^ However, bulk RNA sequencing (RNA‐seq) is insufficient to identify immunological characteristics such as the signalling interactions among immune cells. Furthermore, even if changes at the cellular level have been explored, it focused solely on the local immune response from the gingival tissue.^23^, ^24^ Therefore, to elucidate the systemic immunological relationship between PD and DM, we comparatively analysed peripheral blood mononuclear cells (PBMCs) derived from patients with these diseases using single‐cell RNA‐seq (scRNA‐seq) technology.

## 2 METHODS

### 2.1 Subject recruitment and criteria

Based on the medical history obtained from the recorded questionnaire and interviews, healthy individuals and patients with PD, with or without DM, were included in the study. Individuals with good periodontal health were characterised by the absence of clinically detectable inflammation symptoms, including bleeding on probing, erythema, oedema and bone loss. All PD and both PD and DM (PDDM) patients had stage III periodontal disease. Stage III PD was defined by the deepest interproximal clinical attachment level of 5 mm or more, and its severity was evaluated by an experienced periodontist based on the 2017 World Workshop criteria.^25^ Additionally, all participants were non‐smokers in the year leading up to the screening. Presence of PDDM was verified based on Hb1AC levels and periodontal status. Individuals with systemic illnesses other than DM or those who received periodontal therapy or an antibiotic prescription within 6 months were excluded from the study.

### 2.2 PBMC collection and isolation

Venous blood was drawn by regular venipuncture, and blood samples were collected in plastic tubes containing ethylenediaminetetraacetic acid. The PBMCs were separated using SepMate within 30 min of collection. Density gradient medium was added to the insert, and the SepMate tube was filled with an identical amount of blood samples diluted with phosphate‐buffered saline (PBS) and 2% foetal bovine serum (FBS). The tubes were then centrifuged at 1200 × *g* for 10 min at room temperature. The top layers were transferred to fresh tubes and washed twice with PBS containing 2% FBS. Tubes were centrifuged at 120 × *g* for 10 min at room temperature. The obtained PBMCs were then frozen and stored at −80°C.

### 2.3 Preparation and scRNA‐seq

Libraries for scRNA‐seq were generated using a chromium controller according to the 10× Chromium Next GEM Single Cell 3′ v3.1 protocol. After mixing the cell suspension and loading onto Single Cell 3′ v3.1 Gel Beads, the cDNA library was amplified and sequenced on an Illumina HiSeq platform. Single‐cell gene expression data were processed using the 10× Genomics Cell Ranger v5.0.1 and FASTQ files were generated using ‘cellranger mkfastq’. Raw FASTQ files were analysed using ‘cellranger count’. The reads were aligned to the human reference genome (GRCh38, v3.0.0), and the gene expression matrix was measured using a unique molecular identifier (UMI) and cell barcode.

### 2.4 Preprocessing and normalisation of scRNA‐seq data

The generated scRNA‐seq lists were loaded, merged and preprocessed using Seurat (version 4.1.1).^26^ For each sample, the doublet cells were predicted and removed using DoubletFinder.^27^ The remaining cells were filtered based on read counts, gene numbers and mitochondrial gene counts. Cells with <500 or >15 000 UMIs and with >15% mitochondrial reads were excluded. Cells were kept if they had ≥250 genes and ≤5000. In total, 243 038 cells and 23 492 genes were included in the downstream analysis. The filtered dataset was normalised and integrated with SCTransform, SelectIntegrationFeatures and PrepSCT Integration functions.

### 2.5 Cell identification

Principal component (PC) analysis and visualised uniform manifold approximation and projection were performed on the 20 PCs selected for further study based on their contributions to variability. Based on the 20 PCs, the nearest neighbours were computed, and cells were clustered using the FindClusters function. Marker genes for each cell population were detected using the FindAllMarkers function and defined as differentially expressed genes (DEGs) with an average log fold change >.5 and Bonferroni adjusted *p*‐value <.01. The cell types were identified by referring to multiple immune references that covered the bases of typical PBMCs in SingleR (version 1.6.1).^28^

### 2.6 Analysis of scRNA‐seq data

To compare the transcriptomic characteristics of the three groups (healthy donors, PD and PDDM), differential expression tests were conducted using the FindMarkers function with a two‐sided Wilcoxon rank‐sum test. As this was a multivariate test, DEGs with a Bonferroni adjusted *p*‐value <.01 were considered significant. Overrepresentation analysis was performed to annotate the biological functions of the genes using ClusterProfiler (version 4.0.5). Terms with a *p*‐value <.05, which were enriched in more than three genes, were considered statistically significant.

### 2.7 Preprocessing of public scRNA‐seq datasets

We downloaded the publicly available datasets GSE165816 and GSE164241 from Gene Expression Omnibus (https://www.ncbi.nlm.nih.gov/gds). GSE165816 includes the transcriptional profiles of PBMCs obtained from patients with DM and healthy donors. GSE164241 is a scRNA‐seq dataset of gingival tissues from patients with PD and from healthy controls. The group of patients exhibited moderate to severe periodontal disease, characterised by probing depths exceeding 5 mm in more than four interproximal sites, along with noticeable tissue inflammation, including erythema/oedema and bleeding upon probing. In addition, all individuals were non‐smokers within 1 year of the screening. To integrate with the inclusion criteria used for the in‐house data, patients with DM patients along with morbid obesity, alcohol abuse, as well as pre‐diabetes healthy donors were excluded.

In GSE165816, cells with >15 000 UMIs and >20% of mitochondrial reads were excluded. In GSE164241, cells that expressed <200 genes, >5000 genes and >15% mitochondrial reads were removed as described in a previous study. We then normalised and integrated the samples for each dataset using the SCTransform(), PrepSCTIntegration(), FindIntegrationAnchors() and IntegrateData() functions. Putative immune cell populations were extracted from the gingival tissue and subjected to repeated integration and normalisation processes.

### 2.8 Cell state scoring

We obtained scores using the AddModuleScore function, which averages the gene expression levels of the supplied gene list and subtracts the randomly selected control genes. The cytotoxicity‐related genes were *PRF1*, *IFNG*, *GNLY*, *NKG7*, *GZMB*, *GZMA*, *GZMH*, *KLRK1*, *KLRB1*, *KLRD1*, *CTSW* and *CST7* and the exhaustion‐associated genes were *LAG3*, *TIGIT*, *PDCD1*, *CTLA4*, *HAVCR2* and *KLRG1* for natural killer (NK) and CD8+ T cells.^29^ For scoring NK cell activity, we used the gene list in the Gene Ontology (GO) term GO:0002323, indicating ‘natural killer cell activation involved in immune response’.

### 2.9 Trajectory inference

The Python package VIA was used to determine the stage of CD8+ T and NK cell differentiation.^30^ Each cluster that expressed high levels of CCR7 and SELL in the CD8+ T cell lineage and IL7R in the NK cell lineage was designated as a root cluster. The topology and pseudotimes were constructed using a combination of lazy‐teleporting random walks and Monte‐Carlo Markov chain simulations. The R package DEsingle was used to investigate the DEGs for each cluster in the terminal state.^31^

### 2.10 Inference of cell–cell interaction

The pattern and intensity of the intercellular signalling pathways were predicted using the R package CellChat (version 1.1.3).^32^ A normalisation matrix was loaded and run for each group. We used a built‐in ligand–receptor (LR) interaction database for humans and estimated the probability of communication between cell groups in a specified group. The strength of the interaction was extracted from the inferred interaction weight, and cell chat objects were merged and visualised through a netVisual_heatmap and netVisual_aggregate functions to compare the three groups.

### 2.11 Statistical analysis

The differences among three and two groups were determined using the non‐parametric Kruskal–Wallis rank sum test and a two‐sided Wilcoxon rank‐sum test, respectively. The threshold of significance in the univariate test was set at a *p*‐value of .05.

## 3 RESULTS

### 3.1 scRNA‐seq map landscape of PBMCs

**FIGURE 1:**
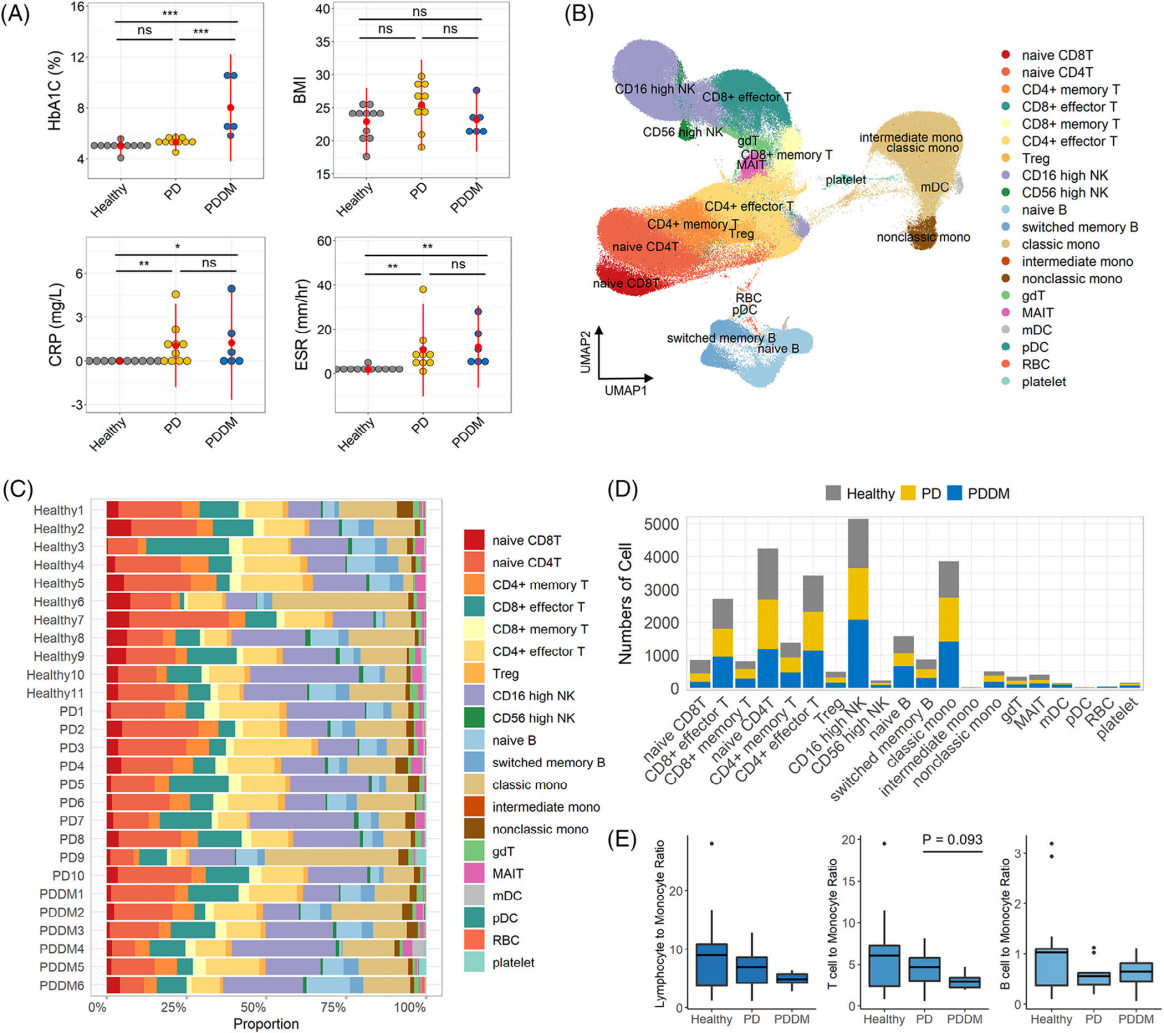
Peripheral blood mononuclear cells (PBMCs) and clinical profiles of healthy donors and of patients with periodontitis (PD) and both PD and diabetes mellitus (DM) (PDDM). (A) Clinical information of each group. Body mass index (BMI), haemoglobin A1c (HbA1c), C‐reactive protein (CRP) and erythrocyte sedimentation rate (ESR) were recorded. Error bars represent mean ± standard deviation (SD). All differences with *p*‐values <.05 are marked: Wilcoxon rank‐sum test. ^*^ *p* < .05, ^**^ *p* < .01, ^***^ *p* < .0005, ns: not significant. (B) Uniform manifold approximation and projection (UMAP) projections of integrated PBMCs. The cell compartments consist of naïve T cells, effector and memory T cells, regulatory T cells (Treg), natural killer (NK) cells, naïve B cells, switched memory B cells, classical monocytes (classic mono), intermediate monocytes (intermediate mono), non‐classical monocytes (nonclassic mono), gamma delta T cells (gdT), mucosal‐associated invariant T cells (MAIT), myeloid dendritic cells (mDC), plasmacytoid DCs (pDC), red blood cells (RBC) and platelets. (C) Bar plot of the immune cell fraction for each individual. (D) Bar plot showing the number of immune cells. Healthy, grey; PD, yellow; and PDDM, blue. (E) Boxplot of the ratio between lymphocyte‐lineage immune cells and monocytes. The left panel shows the ratio of lymphocytes, including B lymphocytes, T lymphocytes, and NK cells to monocytes. The middle panel shows the ratio of T lymphocytes to monocytes and the right panel shows the ratio of B lymphocytes to monocytes. The horizontal line in the boxplot indicates the average ratio for each group.

Our study included samples from 11 healthy donors, 10 patients with PD and six patients with PDDM. We examined the clinical parameters to understand the characteristics of each group (Table S2). First, HbA1c levels were significantly higher only in the PDDM group (Figure 1A). The body mass indices of patients with PDDM remained within the normal range. For C‐reactive protein and erythrocyte sedimentation rate, which are indicators of inflammation, both patients with PD and PDDM exhibited higher levels than those in the healthy donors. This implies that both the PD and PDDM groups retained systemic inflammation, and that in the patients with PDDM in this study, the inflammation was independent of obesity. After processing the scRNA‐seq data, 243 038 cells with an average of 9000 cells per subject were established. We identified 42 clusters and distinguished 20 cell types based on multiple cell‐type reference datasets using previously identified canonical markers (Figures 1B and S2A–C). Lymphocytes accounted for the majority (approximately 80%), followed by monocytes, regardless of clinical status (Figure 1C,D). Although proportional heterogeneity was found among individuals, no significant differences were observed in the cellular fraction depending on the disease status. To estimate inflammation at the cellular level, we calculated the lymphocyte‐to‐monocyte ratio (LMR), which is a negatively correlated inflammatory marker in many diseases, including diabetic kidney injury, colon cancer and lung cancer.^33^, ^34^, ^35^ The LMR was decreased in both PD and PDDM, with the lowest levels observed in PDDM (Figure 1E). Furthermore, the T cell‐to‐monocyte ratio showed a trend similar to that of LMR rather than to that of B cells. This suggests that the systemic immune environment is altered in PD and PDDM, primarily because of T lymphocytes and monocytes.

### 3.2 DM accentuates inflammation by modulating classical monocytes and CD4+ effector T cells

**FIGURE 2:**
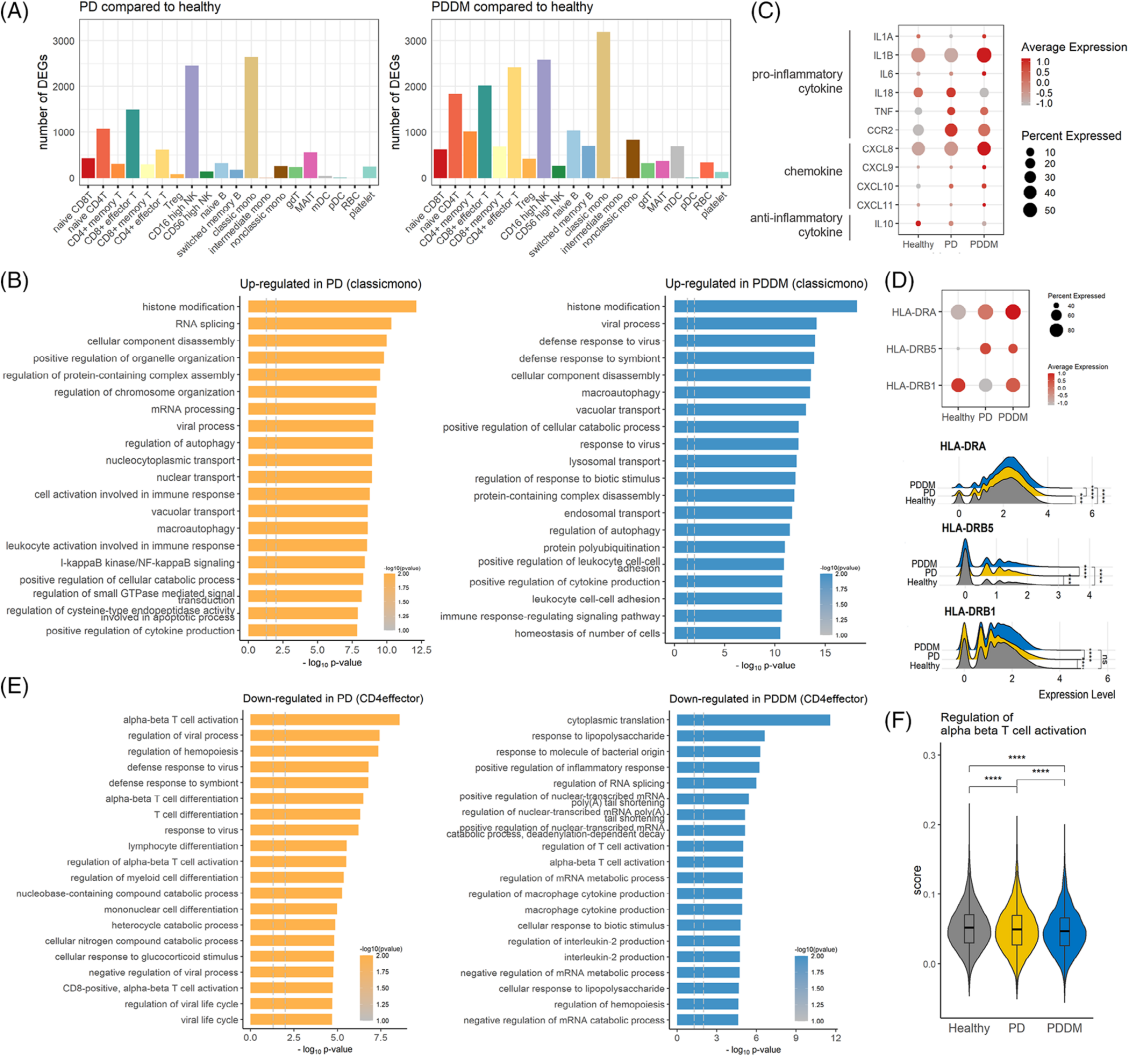
Immunological shifts of monocytes and CD4 effector cells in periodontitis (PD) and both PD and diabetes mellitus (DM) (PDDM). (A) Number of differentially expressed genes (DEGs) in each cellular compartment. Bar plots display the number of DEGs when comparing patients with PD to healthy controls (left) and patients with PDDM to healthy controls (right). (B) The top 20 Gene Ontology (GO) terms of DEGs in classical monocytes. Functions of upregulated genes in patients with PD (left) and PDDM (right) compared with those in the healthy controls are shown. (C) Expression levels of pro‐inflammatory and anti‐inflammatory cytokines and chemokines in monocytes. (D) Expression of human leukocyte antigen‐DR (HLA‐DR). Patients with HLA‐DRA, HLA‐DRB5 or HLA‐DRB1 mutations were included in this study. The dotted plot in the upper panel shows the expression levels of HLA‐DR genes and the ridge plot below shows the distribution of HLA‐DR gene expression. The significance of the differences was computed using the Wilcoxon rank‐sum test. ^*^ *p* < .05, ^**^ *p* < .01, ^***^ *p* < .001, ^****^ *p* < .0001, ns: not significant. (E) The top 20 GO terms of DEGs in CD4+ effector cells. Functions of downregulated genes in PD (left) and PDDM (right) compared to those in the healthy controls are shown. (F) Violin plot showing the CD4+ effector cell activity score regulating alpha–beta T cell activation. The expression levels of genes listed in GO:0046634 (regulation of alpha–beta T cell activation) were measured.

To explore the functions of immune cells in patients with PD and PDDM, we investigated the DEGs for each cell type (Figure 2A and Tables S3 and S3). Both PD and PDDM showed different transcripts across all immune cells. Marked changes were found in the gene expression of classical monocytes in both the PD and PDDM groups. However, unlike patients with PD, patients with PDDM showed prominent changes in CD4+ effector T cells (Figure 2A). Next, we examined the function of classical monocytes and CD4+ effector cells. In the PD and PDDM groups, the classical monocytes showed increased biological processes, such as autophagy, leukocyte activation and positive regulation of cytokine production (Figures 2B and S3A). The PD group expressed higher levels of pro‐inflammatory cytokines, including *IL18*, *TNF* and *CCR2*, than those in the healthy controls (Figure 2C). The PDDM group displayed increased gene expression of all pro‐inflammatory cytokines and chemokines, except for *IL18*. Both patients with PD and PDDM showed decreased levels of *IL10*, an anti‐inflammatory cytokine, with the lowest levels found in patients with PDDM. As important molecules for antigen presentation, the transcript levels of all human leukocyte antigen‐DR subunit‐encoding genes were elevated in the PDDM group (Figure 2D).

In the CD4+ effector T cells, responses to lipopolysaccharides and bacterial origin antigens, as well as T‐cell activation and differentiation and IL‐2 production, were diminished in PDDM (Figures 2E and S3B,C). This raised suspicions regarding the reduced responsiveness of CD4+ effector T cells. We then measured the expression levels of genes listed in GO as ‘GO:0046634 regulation of alpha–beta T cell activation’. The results showed that the CD4+ effector T cells in PDDM demonstrated the greatest reduction in activation, whereas those in PD showed comparatively low activation compared to that under healthy conditions (Figure 2F). Taken together, co‐occurrence of DM impairs CD4+ T cell activation and increases the levels of pro‐inflammatory cytokines in classical monocytes.

### 3.3 Functions of CD8+ effector T and NK cells are influenced by the presence of DM

**FIGURE 3:**
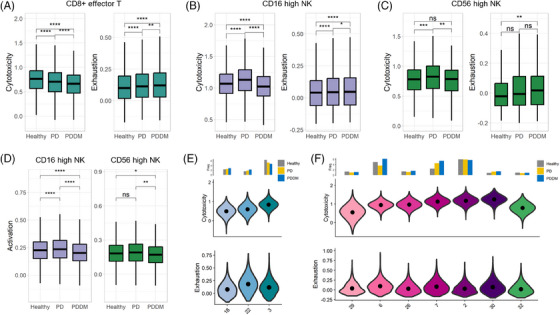
The cytotoxicity, exhaustion and activity scores of CD8 T and NK cells. (A–C) Cytotoxicity and exhaustion of (A) CD8+ effector T, (B) CD16‐high NK and (C) CD56‐high NK cells separated by group. The significance of strength between groups was compared using the Wilcoxon rank‐sum test. ^*^ *p* < .05, ^**^ *p* < .01, ^***^ *p* < .001, ^****^ *p* < .0001, ns: not significant. (D) Activity of CD16‐high and CD56‐high NK cells. Figure descriptions are similar to those of Figure 2A–C. (E) Violin plot of cytotoxicity and exhaustion scores according to clusters, which are identified as CD8+ effector T cells in cell‐type annotation step. The bar plot located above the violin plot shows the proportion of the clusters in each individual group. (F) The violin plot of cytotoxicity and exhaustion scores according to clusters, which are identified as CD16‐high NK cells (clusters 29, 6, 26, 7, 2 and 30) and CD56‐high NK cells (cluster 32) in cell‐type annotation step. The description of the figure is similar to that of Figure 2E.

To understand the effector functions in chronic inflammatory states, we investigated the cytotoxicity and exhaustion of CD8+ effector T and NK cells. CD8+ effector T cells were the most exhausted, whereas CD16‐high NK and CD56‐high NK cells were the most cytotoxic among all immune cell compartments (Figure S3A,B). CD8+ effector T cells exhibited exhaustion in both the PD and PDDM groups, with the PDDM group displaying the greatest exhaustion and lowest cytotoxicity (Figure 3A). The CD16‐high NK and CD56‐high NK cells demonstrated increased cytotoxicity under PD conditions (Figure 3B,C). However, in the PDDM group, cytotoxicity was lower or unchanged compared to that in the healthy group. Additionally, exhaustion was elevated in the PDDM group. As NK cells need to be stimulated before they release toxic granules, we investigated their activation level. Consequently, all NK cells were the least active under the PDDM conditions compared to those under other conditions (Figure 3D). In summary, DM induces functional damage to CD8+ T and NK cells, disrupting the balance of immunological defense.

### 3.4 PD and PDDM show a distinct transition state of CD8+ T and NK cells

**FIGURE 4:**
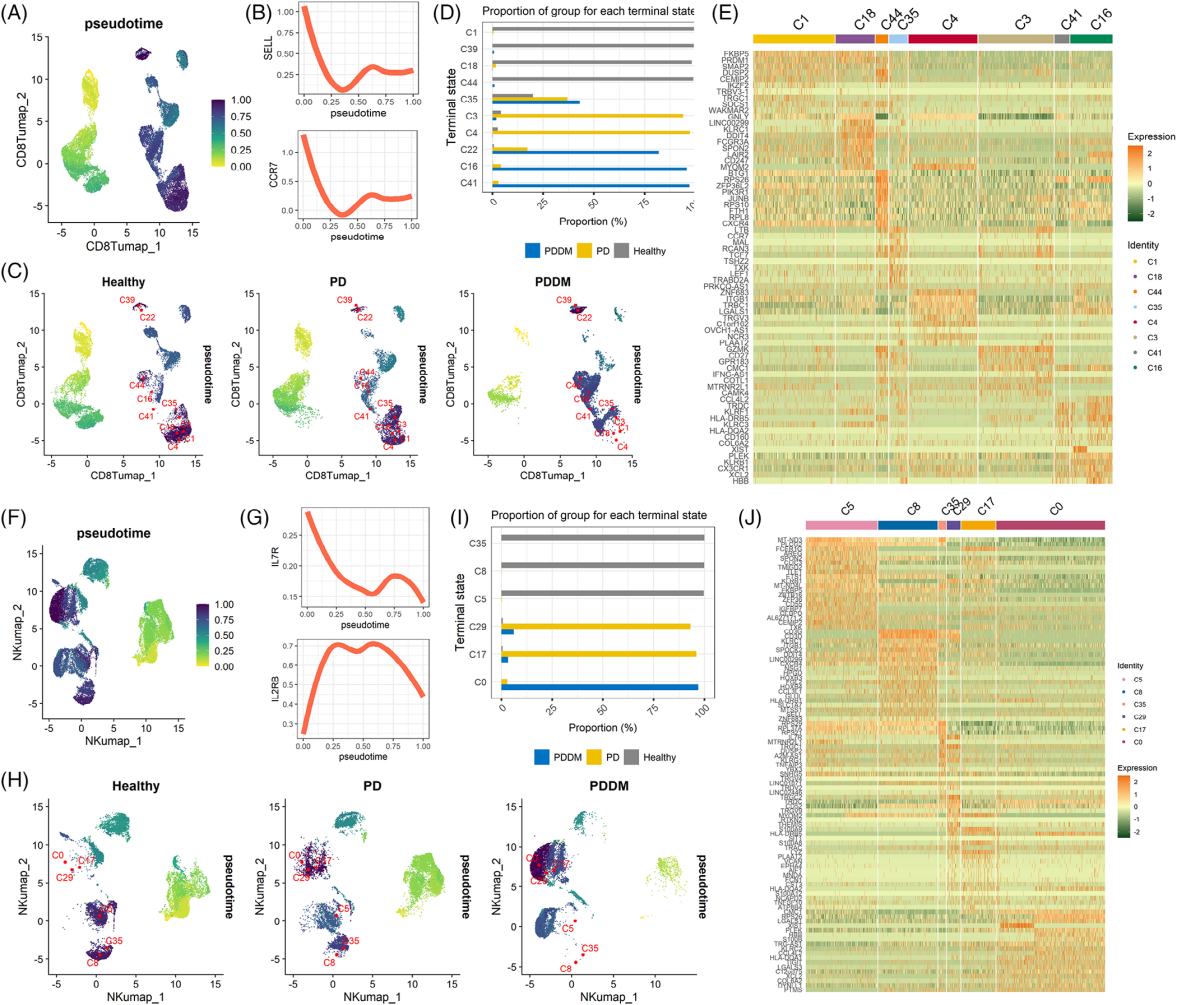
Trajectory analysis of CD8+ T cells and NK cells reveals separated diffusion paths depending on the health conditions. (A) Inferred pseudotime of the CD8+ T‐cell lineage overlaid on uniform manifold approximation and projection (UMAP). CD8+ T cells are coloured according to their pseudotime, with yellow indicating early differentiation and dark blue representing late differentiation. (B) Expression levels of SELL and CCR7, early‐stage markers of CD8+ T cells, ordered by pseudotime. (C) The UMAP was separated by the healthy group. The red text depicts the terminal states detected in the CD8+ T cell lineage pathway. (D) Percentage of cells in each terminal cluster of CD8+ T cells by patient group. (E) Heatmap representing differentially expressed genes for each terminal cluster of CD8+ T cells. The scaled expression levels of extracted genes correspond to the colours depicted in the colour bar. (F) Inferred pseudotime of the NK cell lineage overlaid on the UMAP. NK cells are coloured according to their pseudotime, with yellow indicating early differentiation and dark blue representing late differentiation. (G) Expression levels of diffusion stage markers in NK cells ordered by pseudotime. IL7R is a marker of NK cell precursors, and IL2RB is a marker of NK cell maturation during differentiation. (H) The UMAP was separated by health group. The red text depicts the terminal states detected in the NK cell lineage pathway. (I) The percentages of cells by patient groups for each terminal cluster of NK cells. (J) Heatmap showing differentially expressed genes in each terminal cluster of NK cells. The scaled expression levels of the extracted genes correspond to the colours depicted in the colour bar.

In addition to disease conditions, the subclusters of CD8+ effector cells and NK cells display functional heterogeneity (Figure 3E,F). Therefore, continuously scrutinising the cellular states and determining their response to different environments is necessary. Thus, we performed a trajectory analysis using the VIA package and determined the dynamics of cell differentiation. As the later phase progressed, the expression levels of *CCR7* and *SELL*, which are early‐stage indicators of CD8+ T lineage cells, were diminished (Figure 4A,B). The terminal state was determined based on the vertex connectivity properties of the directed graph (Figures S4 and 4C). Cells of the early CD8+ T‐cell lineage were sparse in PD and PDDM. The high‐density clusters of the later phase also moved in distinct directions according to health status. Among the terminal clusters, C1, C39, C18 and C44 were abundant in healthy individuals, whereas clusters C22, C16 and C41 were enriched in PDDM (Figure 4D). To investigate cluster‐specific attributes, we identified the DEGs for each terminal cluster (Figure 4E). C1 and C18 were characterised by genes related to T cell differentiation, development and T cell antigen receptor signalling cascades, such as *PRDM1*, *DUSP2*, *IKZF2* and *CD247*.^36^, ^37^, ^38^, ^39^ C41 and C16 expressed higher levels of the inhibitory receptor genes *KLRB1*, *CD160* and *CX3CR1*.^40^, ^41^, ^42^, ^43^ Overall, the coexistence of PD and DM modifies the properties of CD8+ T cells in the later phases.

NK cells established a pseudotime matching the expression pattern of the acknowledged NK‐cell developmental markers (Figure 4F,G).^44^ The NK cells of patients with PD attained a state similar to that of the healthy group but did not advance to the final differentiation stage. Their destination was closer to cluster C0, which was exclusively found in PDDM (Figure 4H,I). Healthy individuals predominantly showed clusters C5, C8 and C35. High levels of *PLCG2* and *ARE*, which are required for NK cell cytotoxicity, were expressed in C5 (Figure 4J).^45^, ^46^ However, cluster C0 highly expressed inhibitory receptor *TIGIT*.^47^ Furthermore, *XCL2*, which is constitutively expressed in unstimulated NK cells, was found to be upregulated in cluster C0.^48^, ^49^ Overall, these results indicate that NK cells existing under PDDM conditions are likely to enter an orbit out of normal diffusion, thus rendering them incapable of responding to external stimuli.

### 3.5 Certain interactive pathways are enriched for chronic inflammatory statuses

**FIGURE 5:**
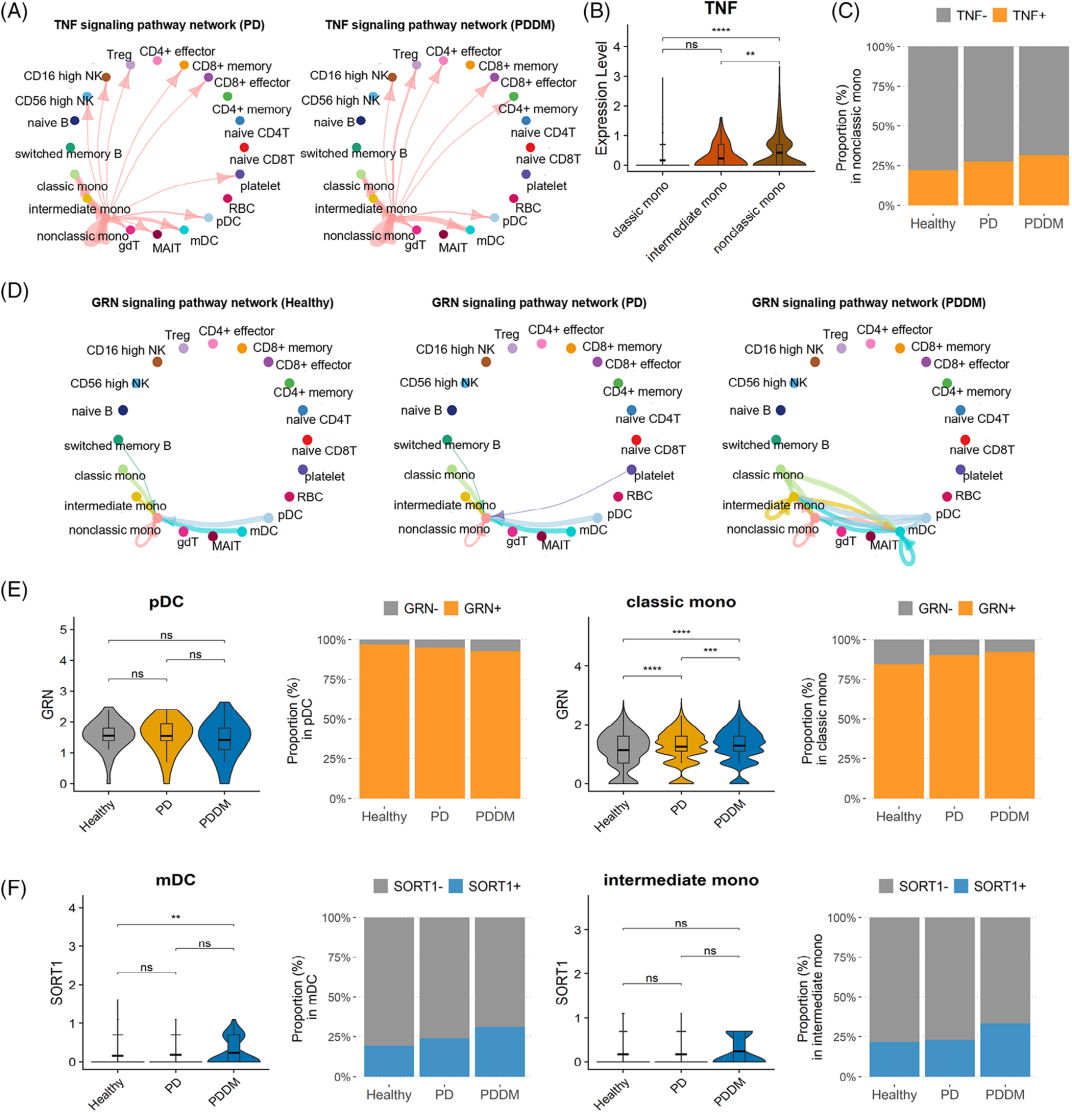
The TNF pathway is strengthened under PD and PDDM conditions whereas the GRN pathway is enhanced under PDDM conditions. (A) Circle plot of TNF signalling in PD and PDDM. The cell–cell crosstalk of TNF signalling in PD is shown in the left panel, and that of PDDM is shown in the right panel. The arrow starts from the signal sender and ends directly at signal receiver. The colour of the arrow matches that of the sender cell. The strength of the crosstalk is correlated with the edge width. (B) Violin plot of TNF expression in monocyte subpopulations. Expression levels between groups were compared using the Wilcoxon rank‐sum test. ^*^ *p* < .05, ^**^ *p* < .01, ^***^ *p* < .001, ^****^ *p* < .0001, ns: not significant. (C) Percentage of TNF+ non‐classical monocytes in the control, PD and PDDM groups. (D) Circle plot of GRN signalling. The cell–cell crosstalk of TNF signalling in healthy, PD and PDDM groups is drawn in the left, middle and right, respectively. (E) GRN expression and percentage of GRN+ cells in the control, PD and PDDM groups. Violin and bar plots in the left panel show the expression level of GRN in pDCs and the proportion of GRN‐expressing pDCs, whereas the right panel shows the GRN expression and proportion of GRN‐expressing classical monocytes. (F) SORT1 expression and percentage of SORT1+ cells in the control, PD and PDDM groups. Violin and bar plots in the left panel show the expression level of SORT1 in mDCs and the proportion of SORT1‐expressing mDCs, whereas the right panel shows SORT1 expression and the proportion of SORT1‐expressing intermediate monocytes.

To better understand the relationship between circulating immune cells and the chronic inflammatory status, we inferred the cell–cell interactions in each condition. Cell–cell communication analysis revealed differentially enriched pathways of TNF, GRN and RESISTIN (Figure S5). TNF signalling, involving the TNF–TNFRSF1A and TNF–TNFRSF1B LR pairs, was strongly activated both in PD and PDDM (Figures 5A and S6A). Non‐classical monocytes were the only cells sending signals and expressed highly increased levels of *TNF* among monocyte subpopulations (Figure 5A,B). The ratio of *TNF*‐expressing non‐classical monocytes was augmented under PD and PDDM conditions (Figure 5C). Thus, non‐classical monocytes contribute to the generation of TNF signals under both chronic inflammatory conditions.

The GRN signal, which affects wound healing, inflammation and tumourigenesis, was detected only in PDDM.^50^ The healthy controls and patients with PD showed comparable communication networks; however, the PDDM group exhibited more intense and diverse cell engagement (Figure 5D). The GRN–SORT1 axis was primarily observed in myeloid cells, with enhanced signals in pDC and classical monocytes in PDDM (Figure S6B). Furthermore, mDCs and intermediate monocytes were involved as signal receivers. Classical monocytes showed significantly elevated levels of *GRN* expression in PDDM, whereas mDCs exhibited higher expression levels of the receptor gene *SORT1* (Figure 5E,F). Taken together, the strengthened GRN intercellular pathway in PDDM is likely derived from the interactions between classical monocytes and mDCs.

### 3.6 Insulin resistance pathway is intensified in both PD and PDDM

**FIGURE 6:**
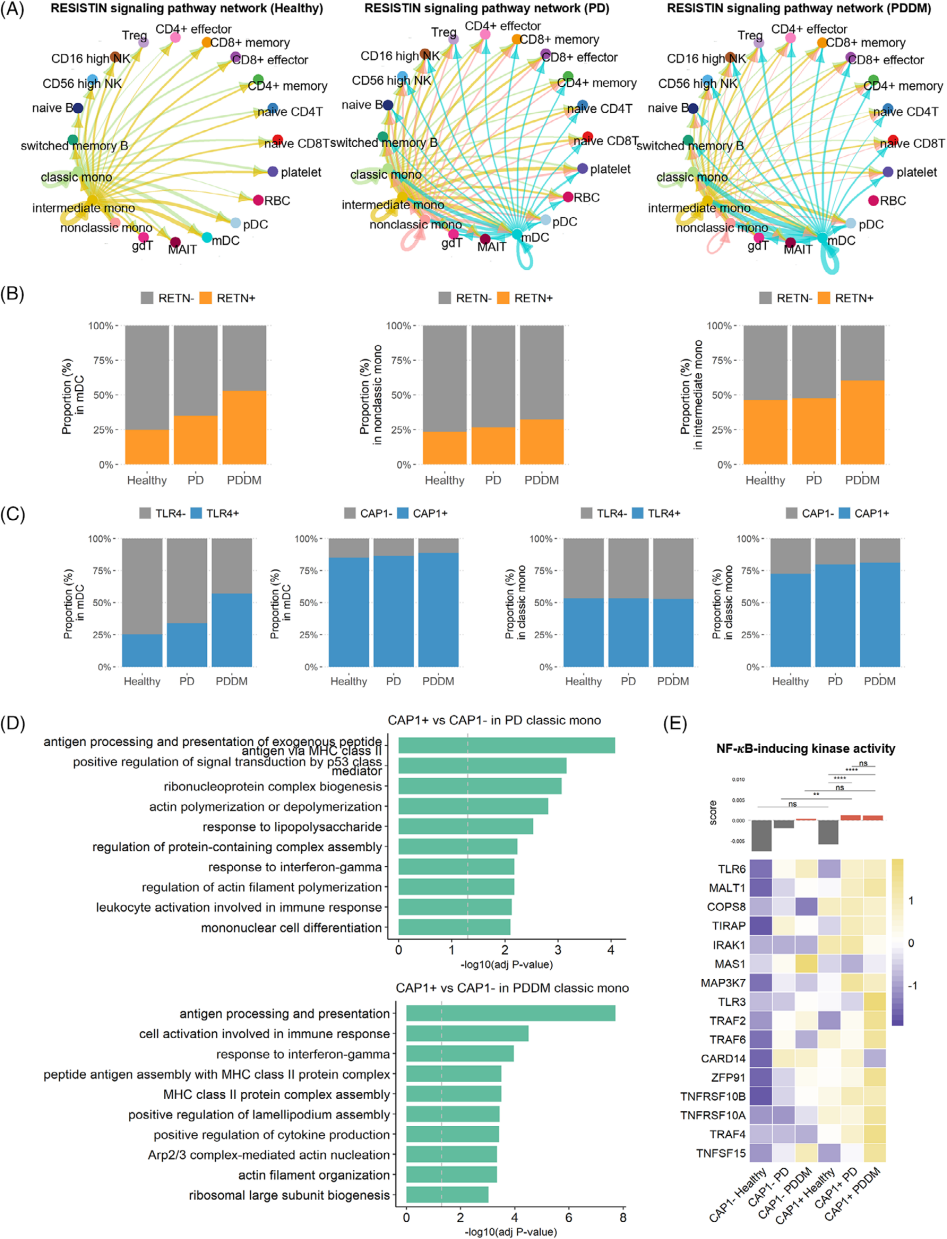
The RESISTIN pathway is intensified in both PD and PDDM, sequentially. (A) Circle plot of RESISTIN signalling. The RESISTIN network in the healthy, PD and PDDM groups are drawn in the order: left, middle and right, respectively. (B) Percentage of ligand‐expressing cells in the RESISTIN pathway in the control, PD and PDDM groups. The bar plots represent the ratio of RETN+ cells in mDCs, and in non‐classical and intermediate monocytes, which play a role in signalling, in that order. (C) Percentage of cells expressing the receptors of the RESISTIN pathway in the control, PD and PDDM groups. Each panel shows the proportion of TLR4+ and CAP1+ cells among mDCs and classical monocytes. (D) Differential functions between CAP1+ and CAP1– classical monocytes. The upper panel depicts the augmented biological functions of CAP1+ classical monocytes in the PD group, whereas the lower panel shows the same functions in the PDDM group. (E) The activity score of NF‐κB‐inducing kinase and the relative expression of genes associated with this activity (GO:0007250) were categorised according to each group. The score was calculated at the single‐cell level, and the expression levels between the two groups were compared using the Wilcoxon rank‐sum test. ^*^ *p* < .05, ^**^ *p* < .01, ^***^ *p* < .001, ^****^ *p* < .0001, ns: not significant.

The RESISTIN pathway enhances insulin resistance and diabetes susceptibility. This pathway comprises the RETN–CAP1 and RETN–TLR4 axes (Figure S6C). The strength of this pathway was enhanced in the PD and PDDM groups, with a sequential increase (Figure 6A). Notably, identical immune cells participate in both PD and PDDM. The mDCs, non‐classical monocytes, and intermediate monocytes strongly stimulate immune cells in PD and PDDM, whereas mDCs and classical monocytes receive more signals (Figure 6B,C).

Among receiver cells, classical monocytes showed increased expression of the *CAP1* receptor, as well as a higher rate of *CAP1*+ cells (Figures 6C and S7). We explored the functions of *CAP1*+ classical monocytes compared to those of *CAP1*– cells using GO analysis (Figure 6D). *CAP1*+ classical monocytes in patients with PD showed higher expression during antigen presentation, response to lipopolysaccharide, leukocyte activation and cell differentiation. Similar pathways were also increased in *CAP1*+ classical monocytes from patients with PDDM, potentially inducing an inflammatory response. As RESISTIN directly binds to CAP1 receptor and enhances inflammatory cytokines via nuclear factor kappa B (NF‐κB)‐dependent pathways, we explored the expression of genes involved in NF‐κB‐inducing kinase activity (GO:0007250).^51^, ^52^ To assess the NF‐κB‐inducing activity levels within individual classical monocytes, we scored the activity based on the expression levels of randomly selected control genes. We also visualised the expression levels of genes included in this pathway (Figure 6E). Consequently, classical monocytes exhibited high NF‐κB‐inducing activity in *CAP1*– PDDM, *CAP1*+ PD and *CAP1*+ PDDM. Moreover, several genes contributing to NF‐κB‐inducing kinase activity showed elevated expression levels in both *CAP1*+ PD and *CAP1*+ PDDM. These findings suggest that PD, with or without DM, involves a pathway that renders patients more susceptible to insulin resistance, specifically through *CAP1*+ classical monocytes.

### 3.7 Comparison across public scRNA‐seq datasets

**FIGURE 7:**
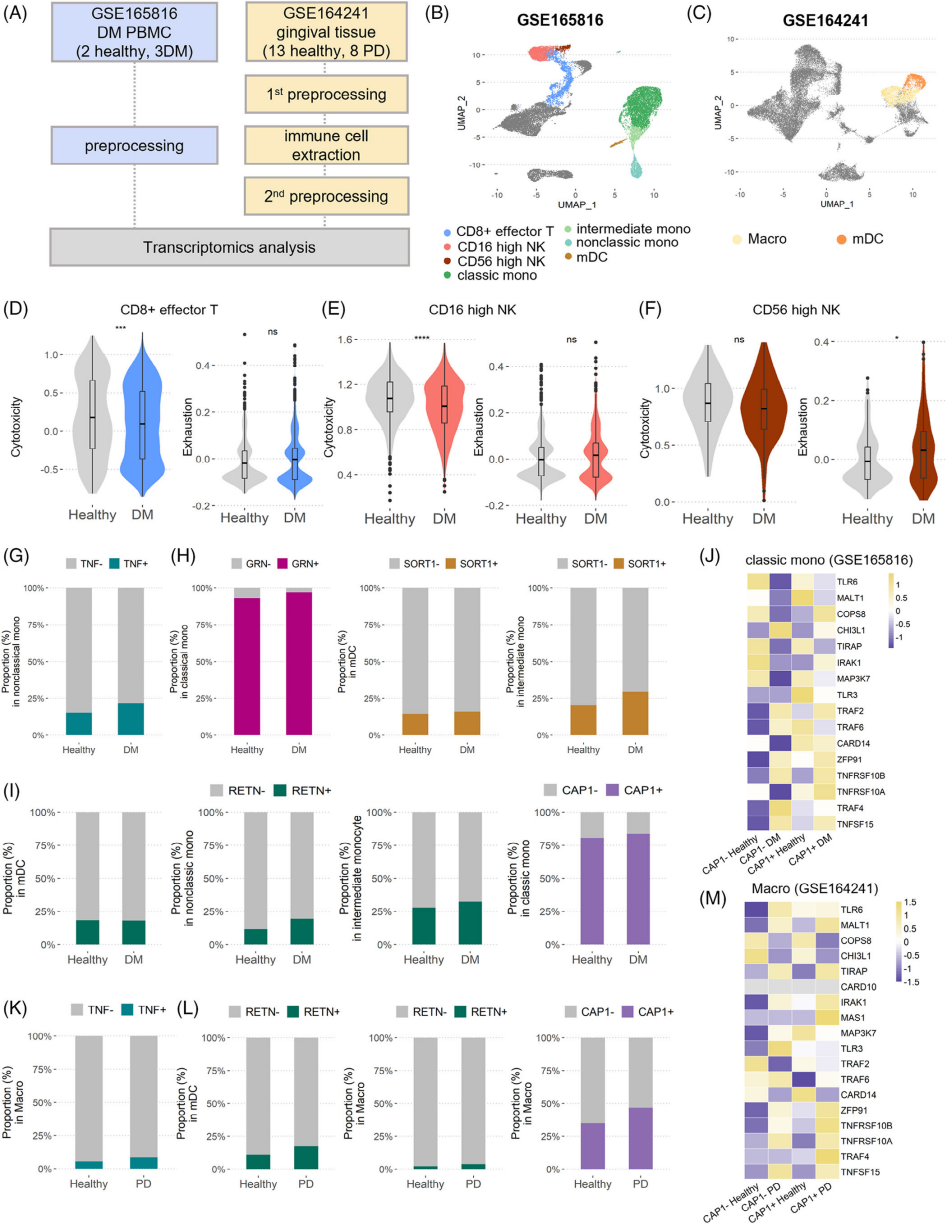
Comparison with publicly available datasets GSE165816 and GSE164241. (A) Summary of the origins of public data samples. (B) Uniform manifold approximation and projection (UMAP) of GSE165816. CD8+ effector T, CD16‐high NK and CD56‐high NK cells; classical, intermediate and non‐classical monocytes; and mDCs were identified for comparison with prior observations. (C) UMAP of GSE164241. Classical monocytes and mDCs were identified for comparison with prior observations. (D–F) Cytotoxicity and exhaustion score of (D) CD8+ effector T cells, (E) CD16‐high NK cells and (F) CD56 high‐NK cells derived from GSE165816. Significant differences between groups were determined using the Wilcoxon rank‐sum test. ^*^ *p* < .05, ^**^ *p* < .01, ^***^ *p* < .001, ^****^ *p* < .0001, ns: not significant. (G) Proportion of TNF+ non‐classical monocytes derived from GSE165816. (H) Proportions of cells expressing the ligands and receptors involved in the GRN pathway (GSE165816). We examined GRN+ classical monocytes, SORT1+ mDCs and SORT1+ intermediate monocytes. (I) Proportion of cells expressing the ligands and receptors involved in the RESISTIN pathway (GSE165816). RETN+ mDCs, RETN+ non‐classical monocytes, RETN+ intermediate monocytes and CAP1+ classical monocytes were examined. (J) Heatmap of gene expression involved in the NF‐κB‐inducing kinase activity within classical monocytes (GSE165816). The cells were separated into the following four groups based on CAP1 expression and health conditions: CAP1– healthy, CAP1– diabetes mellitus (DM), CAP1+ healthy and CAP1+ DM. (K) Proportion of TNF+ macrophages derived from GSE164241. (L) Proportion of cells expressing the ligands and receptors involved in the RESISTIN pathway (GSE164241). RETN+ mDCs, RETN+ macrophages and CAP1+ macrophages were investigated. (M) Heatmap of gene expression involved in the NF‐κB‐inducing kinase activity within macrophages (GSE164241). Macrophages were separated into the following four clusters based on CAP1 expression and health conditions: CAP1– healthy, CAP1– periodontitis (PD), CAP1+ healthy and CAP1+ PD.

We next aimed to confirm our findings using a publicly accessible scRNA‐seq dataset (Figure 7A). Two gene expression datasets (accession numbers: GSE165816 and GSE164241) were retrieved from the GEO database. GSE165816 contains the transcriptomic data of PBMCs sourced from healthy donors and from patients diagnosed with DM (Figures 7B and S8). Meanwhile, GSE164241 contains the transcriptomes of gingival tissues obtained from healthy individuals and from patients with PD (Figures 7C and S9A–C).

As described previously, NK cell subtypes differed between the PD and PDDM groups, whereas CD8+ effector T cells exhibited comparable cytotoxic and exhausted features in both groups. To investigate whether the properties identified in the PDDM group were specific to DM or comorbid conditions, we examined PBMCs from patients with DM alone. The findings indicated that individuals with DM had a reduced cytotoxic score and a heightened level of exhaustion in both CD8+ effector T cells and CD16‐high NK cells (Figure 7D,E). CD56‐high NK cells in the DM group exhibited increased exhaustion rather than cytotoxicity compared to those in the healthy group, consistent with previous observations in the PDDM group (Figure 7F). Next, we explored the ratios of cells expressing genes associated with enhanced cell–cell interactions in PDDM. In DM PBMCs, we observed an increased proportion of *TNF*+ non‐classical monocytes, as well as ligand *GRN*+ classical monocytes, *SORT1*+ mDCs and *SORT1*+ intermediate monocytes (Figure 7G,H). Within the RESISTIN pathway, there was a proportional increase in the levels of *RETN* ligand and *CAP1* receptor (Figure 7I). Additionally, DM *CAP1*+ classical monocytes were confirmed to express the highest level of genes included in NF‐κB‐inducing kinase activity (Figure 7J). This suggests that the attributes identified in the leukocytes from patients with PDDM closely align with the properties of leukocytes in DM.

Using GSE164241, we examined the ratio of cells expressing genes for TNF and RESISTIN signals. The findings revealed an increase in the proportions of *TNF*+ macrophages, *RETN*+ mDCs, *RETN*+ macrophages and *CAP1*+ macrophages within the gingival tissue. Furthermore, the genes involved in NF‐κB kinase activity also showed high expression levels in *CAP1*+ classical monocytes in PD gingiva. Further investigation suggested that *CAP1*+ macrophages derived from circulating *CAP1*+ monocytes also induce tissue inflammation.

## 4 DISCUSSION

DM regulates glucose levels and causes overall health issues.^2^ One of the most common complications of DM is PD, which is affected by multiple factors, including *Porphyromonas gingivalis*. Lipopolysaccharides in the cell walls of Gram‐negative bacteria trigger inflammatory mediators under hyperglycaemic conditions.^4^, ^10^, ^12^, ^14^, ^15^ Although PD is initiated in local periodontal tissue, inflammatory mediators can affect the whole body by circulating in blood. This can lead to changes in metabolism and inflammation, thereby predisposing patients with PD to DM.^12^

Our findings indicate that variation in T lymphocyte counts reflects the LMR trend better than that of the variation in B lymphocyte counts in both disease groups. As previously reported, T cell assembly is impaired by chronic exposure to inflammatory cytokines.^53^ Furthermore, the number of DEGs was predominantly altered in T lymphocytes. CD4+ effector T cells, which show significant expression changes in patients with PDDM, show reduced activation and a poor response to lipopolysaccharide under PDDM conditions. Similarly, CD8+ T cells exhibit alterations in functions, including cytotoxicity, exhaustion and differentiation. The later phase of CD8+ T cells in the PDDM group showed higher levels of inhibitory receptor genes such as *KLRB1*, *CD160* and *CX3CR1*,^36^, ^37^, ^38^, ^39^ but lower levels of T cell development and antigen receptor signalling genes such as *PRDM1*, *DUSP2*, *IKZF2* and *CD247*.^40^, ^41^, ^42^, ^43^ These results imply that T cell lineages are primarily affected by PD and DM.

Next, we discovered unique alterations found only in PDDM environments. First, NK cell activation showed contrasting patterns in the PD and PDDM groups. NK cells from the PDDM group showed low activity and high exhaustion, whereas those from the PD group showed high activity and cytotoxicity. This closely corresponds with previous results, indicating that the presence of DM leads to reduced NK cell activity and cytotoxicity.^54^, ^55^, ^56^ Specifically, the differentiation pathways of NK cells in PD and PDDM were directed towards a similar destination; however, NK cell activity in the PDDM group was considerably impaired. The later phase of NK cells in the PDDM group showed low expression of *PLCG2* and *ARE*, which are required for NK cell‐mediated cytotoxicity and are secreted by NK cells to promote the growth of normal epithelial cells.^45^, ^46^ In contrast, this population expresses high levels of *TIGIT* and *XCL2*. Deficiency of *TIGIT* in NK cells has been reported to reverse their exhaustion whereas *XCL2* is constantly expressed in unstimulated NK cells.^47^, ^48^, ^49^ Second, the progranulin (GRN) pathway, known to play a role in insulin resistance, was enriched in the PDDM group.^57^ SORT1 is implicated as a receptor in the GRN signalling pathway and plays a role in regulating glucose and lipid metabolism. Studies on SORT1 knockout mice have shown that they exhibit resistance to obesity and increased insulin sensitivity.^58^, ^59^ Our results showed that patients with PDDM showed elevated expression of *GRN* in classic monocytes and *SORT1* in mDCs. Considering that circulating GRN was markedly higher in the DM group compared to that in the control group, the GRN–SORT1 axis may contribute to the dysregulation of glucose control in the bloodstream.^57^, ^60^, ^61^

Notably, patients with PD and those with PDDM share several common intercellular pathways. One of them, the RESISTIN pathway, was augmented in both PD and PDDM compared to that in healthy individuals, with sequential increments. RESISTIN, standing for ‘resistance to insulin’, is an important pro‐inflammatory molecule that affects many chronic inflammatory diseases and is linked with DM.^62^ In PBMCs from the DM group, the ratio of cells expressing the ligand RESISTIN and the receptor CAP1 was elevated. This increase was consistently observed in both PD and PDDM groups. Considering that serum RESISTIN levels in both the PD and PDDM groups were higher than those in the healthy group, RESISTIN interaction is likely to occur in the bloodstream.^63^, ^64^ Remarkably, CAP1+ classical monocytes have shown intensified antigen processing and presentation functions as well as NF‐κB‐inducing activity compared to that of CAP1– classical monocyte in both PD and PDDM conditions. NF‐κB‐inducing activity was also detected in macrophages residing in tissues. Furthermore, the upregulation of pro‐inflammatory cytokines in classical monocytes of both PD and PDDM groups, suggests that the inflammation triggered by this signal could increase the chances of PD progressing to pre‐diabetes.

This study had some limitations. PBMC samples were cryopreserved to select samples from individuals who met the subject criteria; however, this could have potentially affected the integrity of certain cell types. Although T cells from cryopreserved PBMCs have been suggested to recover and maintain viability over a long term, the number of monocytes was reduced compared to that in freshly isolated PBMCs.^65^ Therefore, confirmation of these results using freshly isolated PBMCs is necessary. Second, this study focused on the transcriptome of PBMCs, which excluded neutrophils. Neutrophils are the most prevalent leukocyte population within the periodontal pocket and inflamed periodontal tissues.^66^ Accumulation of bacterial biofilms results in increased inflammation and influx of neutrophils, which can contribute to tissue damage when present in excess.^67^ Although direct evidence remains to be presented, defects in neutrophil chemotaxis, phagocytosis and microbicidal function have been confirmed in patients with DM.^68^ As neutrophils may serve as major mediators in PD and DM, further studies on neutrophils are required. In contrast, *P. gingivalis* has been demonstrated to evolve its pathogenicity by altering the host immune response and microbial community such that a favourable inflammatory environment is established.^69^, ^70^ Notably, *P. gingivalis* colonisation depends on its interactions with the oral microbiome. Therefore, studying the immunological responses in conjunction with the oral microbiome could enhance our understanding regarding the progression from PD to DM.

## 5 CONCLUSIONS

Overall, our study provides the first immunological explanation for the intricate association between PD and DM at the systemic and cellular levels. Our findings elucidate the alteration in immune cell function induced by PD and PDDM and suggest that PD may serve as a precursor to DM through the RESISTIN pathway.

## AUTHOR CONTRIBUTIONS

Yun Hak Kim and Hae Ryoun Park designed and supervised the study. Hansong Lee and Yun Hak Kim performed computational analyses. Ji‐Young Joo, Jae‐Min Song, Hyun‐Joo Kim and Hae Ryoun Park collected patient consent for the study and samples. Hansong Lee, Yun Hak Kim and Hae Ryoun Park discussed the results and prepared the manuscript for publication. All authors have read and approved the final version of the manuscript.

## CONFLICT OF INTEREST STATEMENT

Patents pending by the authors and their institutions.

## ETHICS STATEMENT

The participants provided verbal and written consent regarding the use of their blood samples. The Institutional Review Board of Pusan National University Dental Hospital approved the informed consent of the individuals (IRB No. PNUDH‐2020‐032).

## CONSENT FOR PUBLICATION

Not applicable to individual's personal data.

## Supporting information

Supporting information

Supporting information

Supporting information

## Data Availability

The datasets supporting the conclusions of this study are available from the GEO repositor with accession number GSE244515.
