# Supplementary material for: Immunological link between periodontitis and type 2 diabetes deciphered by single‐cell RNA analysis
Source: Clin Transl Med. 2023 Dec 11;13(12):e1503. doi: 10.1002/ctm2.1503 (PMC10713875; doi:10.1002/ctm2.1503)
Supplement: Supplementary file 3 — Supporting information [file CTM2-13-e1503-s001.docx]

**Supporting Information**

Immunological link between periodontitis and type 2 diabetes deciphered by

single-cell RNA analysis

Hansong Lee^[[1]](#footnote-1)^, Ji-Young Joo^[[2]](#footnote-2)^, Jae-Min Song^[[3]](#footnote-3)^, Hyun-Joo Kim ^[[4]](#footnote-4)^ ^[[5]](#footnote-5)^ ^[[6]](#footnote-6)^, Yun Hak Kim 6 ^[[7]](#footnote-7)^ ^[[8]](#footnote-8)^, Hae Ryoun Park 5 6 ^[[9]](#footnote-9)^

Correspondence to: Yun Hak Kim mail to yunhak10510@pusan.ac.kr and

Hae Ryoun Park mail to parkhr@pusan.ac.kr

| **Contents** |
| --- |
| Table S1. Clinical measurements of subjects |
| Figure S1. Identification of PBMCs using cell type reference datasets and known canonical markers |
| Figure S2. Gene ontology of classical monocytes and CD4+ effector cells |
| Figure S3. Cytotoxicity and exhaustion score of immune cells |
| Figure S4. Clusters on the UMAP space used for inferring the differentiation process |
| Figure S5. Bar plot for comparing cell-cell interaction pathways |
| Figure S6. Bar plot of relative contribution |
| Figure S7. TLR4 and CAP1 expression in receptor-expressing mDCs and classical monocytes for the RESISTIN pathway. |
| Figure S8. Feature plots of immune cell type annotation for DM PBMCs (GSE165816) |
| Figure S9. Macrophage and mDC annotation for PD gingiva (GSE164241) |

**Table S1. Clinical measurements of subjects**

|  | Healthy | PD | PDDM |
| --- | --- | --- | --- |
| Total (n) | 11 | 10 | 6 |
| Female (n) | 4 | 3 | 2 |
| Male (n) | 7 | 7 | 4 |
| age | 53.84 ± 24.26 | 66.47 ± 8.34 | 54.39 ± 8.09 |
| HbA1C (%) | 5.01 ± 0.38 | 5.32 ± 0.37 | 8.01 ± 2.10 |
| BMI | 22.89 ± 2.55 | 25.44 ± 3.42 | 23.18 ± 2.42 |

**Figure S1. Identification of PBMCs using cell type reference datasets and known canonical markers**

**
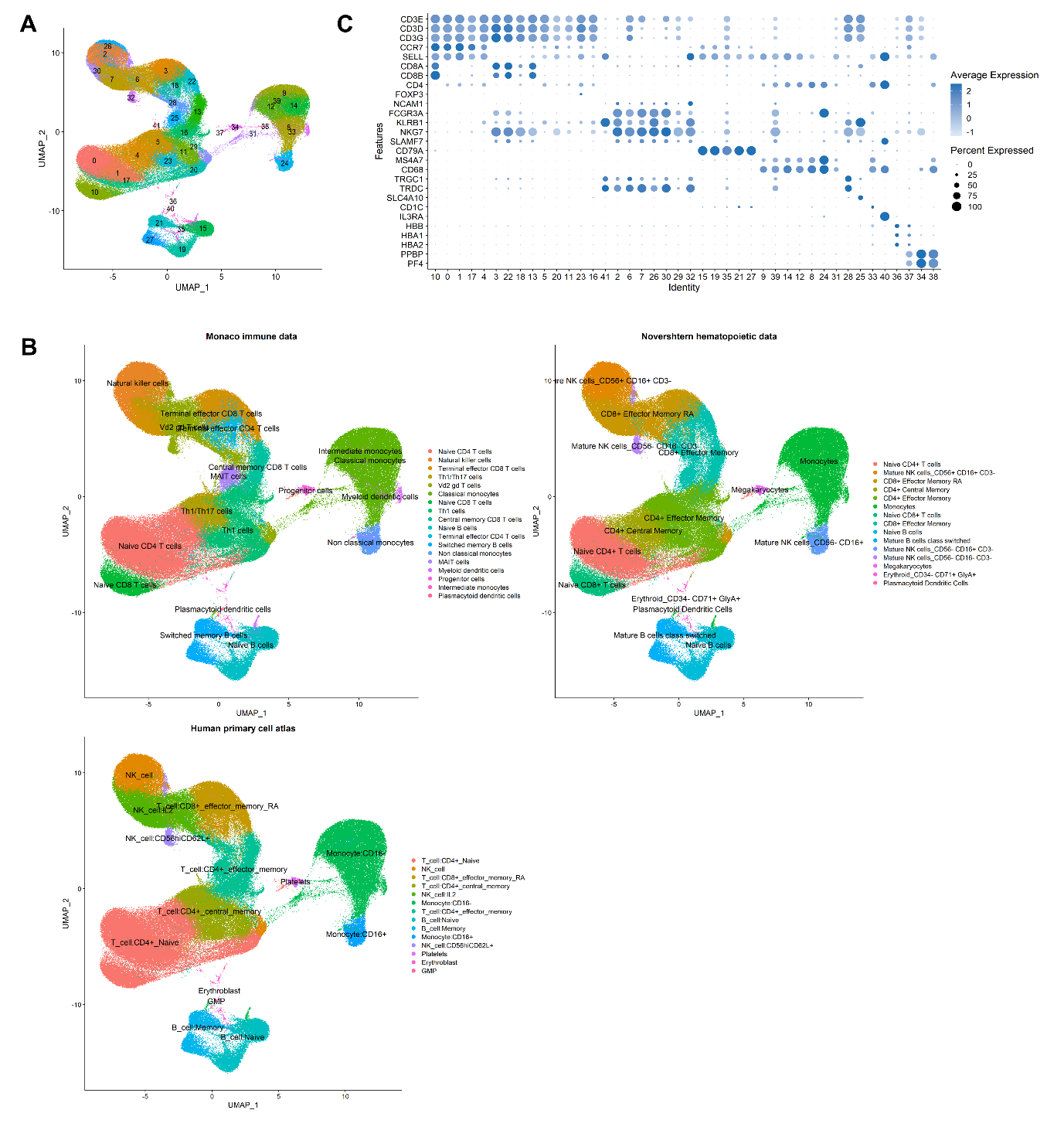
**

A. The 42-population UMAP of PBMCs

B. Cell type identification using three immune cell references. Cell types annotated from the Monaco immune reference, Novershtern reference, and Human Primary Cell Atlas (HPCA) are described.

C. Dot plots of known canonical markers. T cells (CD3E, CD3D, CD3G), naive T cells (CCR7, SELL), CD8 T cells (CD8A, CD8B), CD4 T cells (CD4), Tregs (FOXP3), NK cells (NCAM1, FCGR3A, KLRB1, NKG7), B cells (SLAMF7, CD79A), Monocytes (MS4A7, CD68), gdT (TRGC1, TRDC), MAIT (SLC4A10), mDCs (CD1C), pDCs (IL3RA), RBCs (HBB, HBA1, HBA2), platelets (PPBP, PF4).

**Figure S2. Gene ontology of classical monocytes and CD4+ effector cells**

**
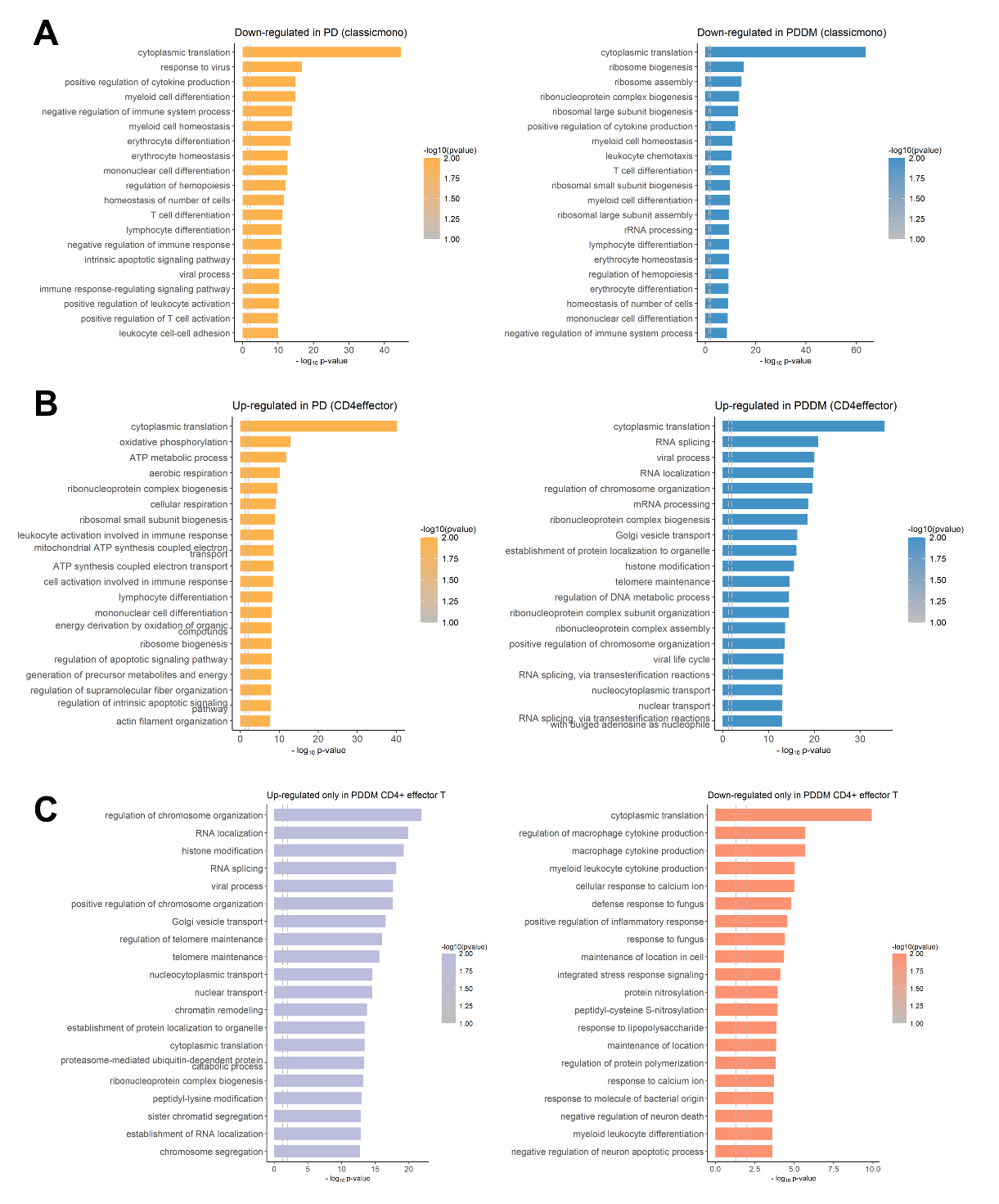
**

1. Biological function of genes downregulated in classical monocytes of PD and PDDM groups compared to those in the healthy controls.
2. Biological function of upregulated genes in CD4+ effector cells of PD and PDDM groups compared with those in the healthy controls.
3. Biological functions of genes differentially expressed exclusively in CD4+ effector cells of the PDDM group compared with those of the PD group and healthy controls.

**Figure S3. Cytotoxicity and exhaustion score of immune cells**

**
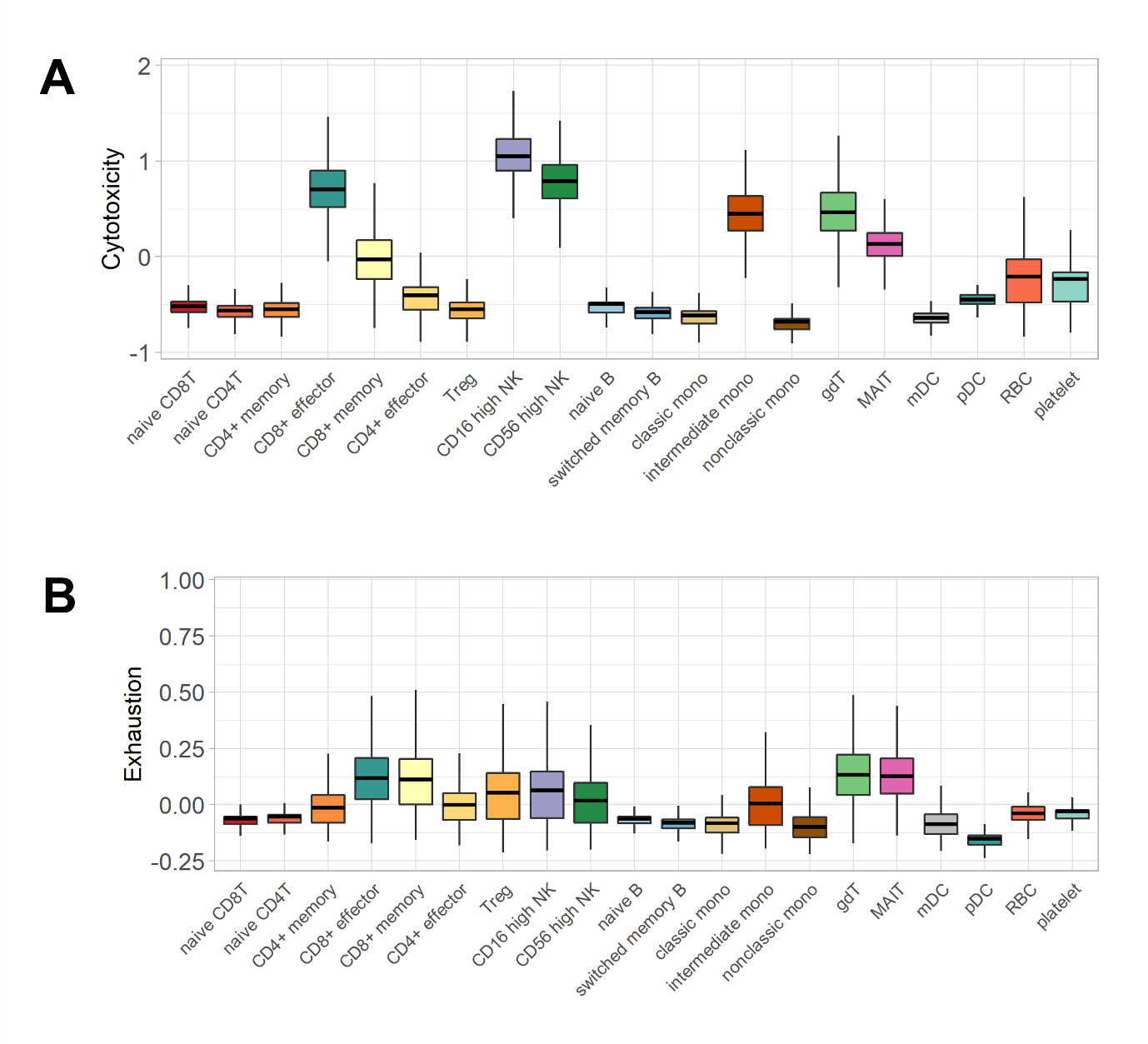
**A. Box plots showing immune cell cytotoxicity PRF1, IFNG, GNLY, NKG7, GZMB, GZMA, GZMH, KLRK1, KLRB1, KLRD1, CTSW, and CST7 were included for scoring.

B. Box plots showing immune cell exhaustion. The genes encoding LAG3, TIGIT, PDCD1, CTLA4, HAVCR2, and KLRG1 were also included.

**Figure S4. Clusters on the UMAP space used for inferring the differentiation process**

**
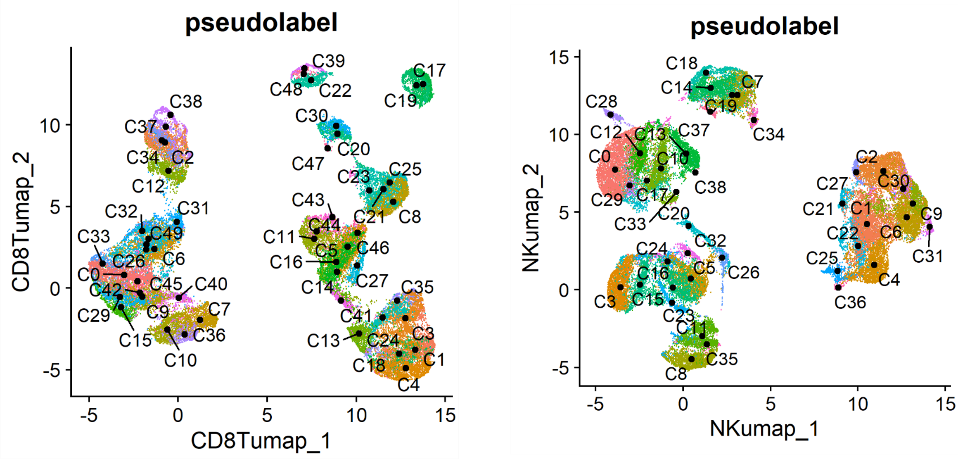
**

Clusters were generalized using the VIA package for CD8+ T and NK cells.

**Figure S5. Bar plot for comparing cell-cell interaction pathways**

**
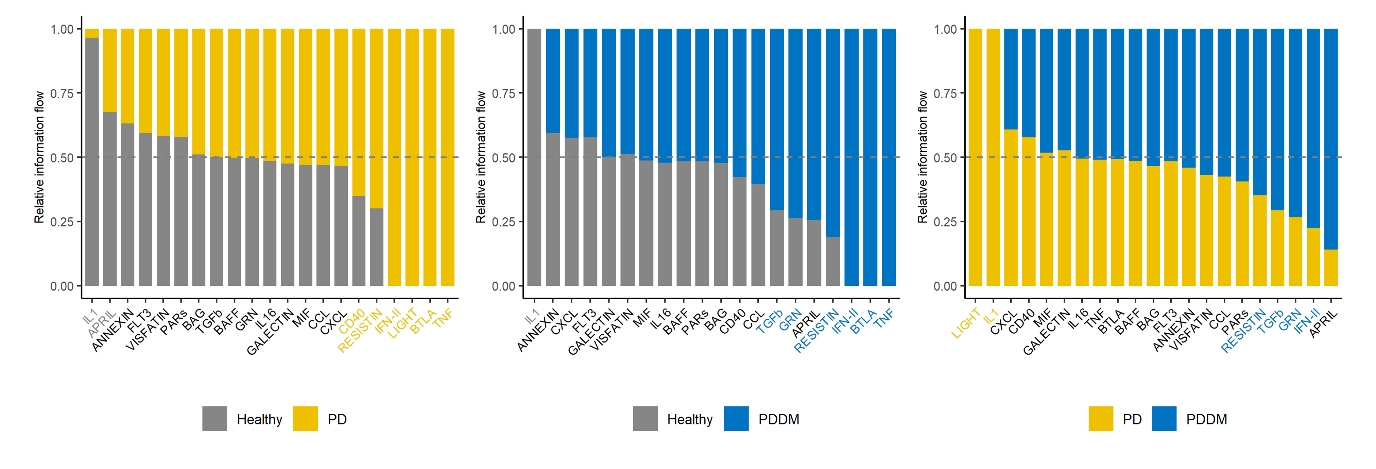
**

Inferenced relative information flow of signaling pathways. The colors of the pathways indicate enriched signals under particular conditions: healthy control (gray), PD (yellow), and PDDM (blue).
 Signals with no significantly different contributions are shown in black. The left panel compares the communication probabilities of healthy controls and patients with PD, Middle panel shows the probabilities of healthy controls and patients with PDDM, and the right panel shows the probabilities of patients with PD and PDDM.

**Figure S6. Bar plot of relative contribution**

**
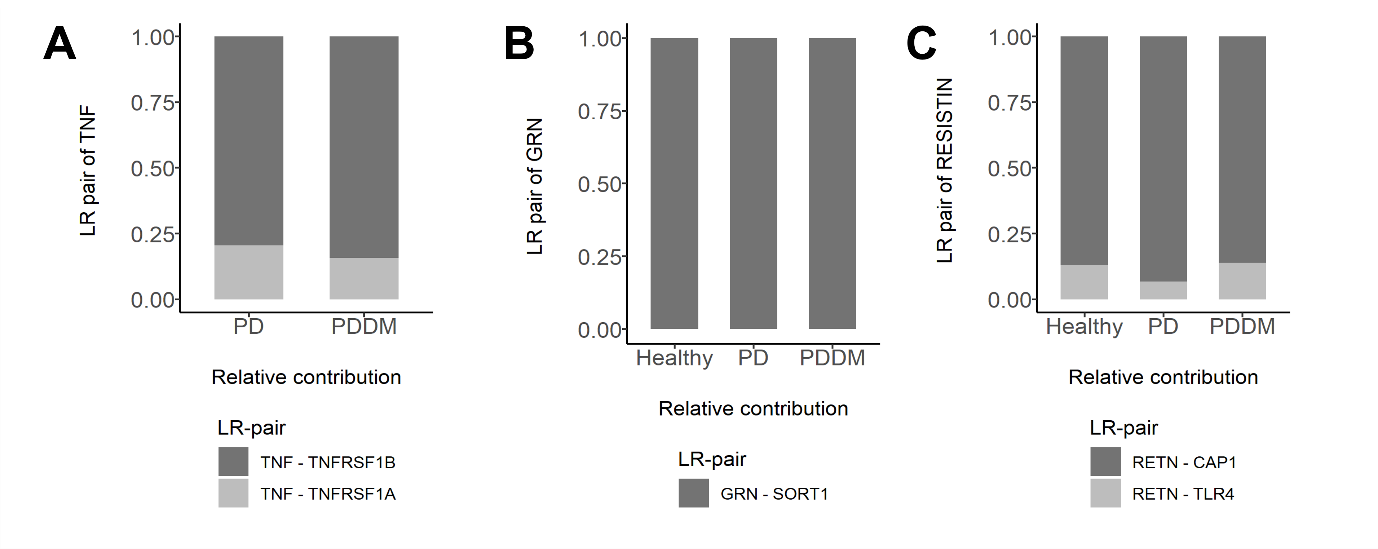
**

1. Contribution of the LR pair to TNF signaling. TNF signaling was not detected in the healthy group.
2. Contribution of the LR pair in GRN signaling.
3. Contribution of the LR pair in RESISTIN signaling.

**Figure S7. TLR4 and CAP1 expression in receptor-expressing mDCs and classical monocytes for the RESISTIN pathway**

**
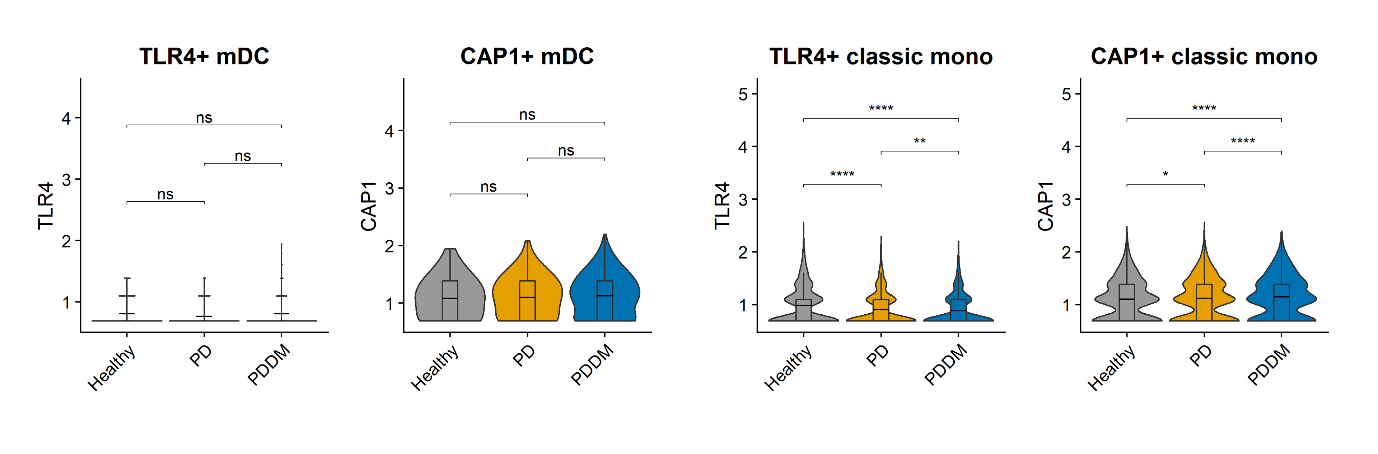
**

TLR4 and CAP1 expression levels in TLR4+ or CAP1+ mDCs and classical monocytes

**Figure S8. Feature plots of immune cell type annotation for DM PBMCs (GSE165816)**

**
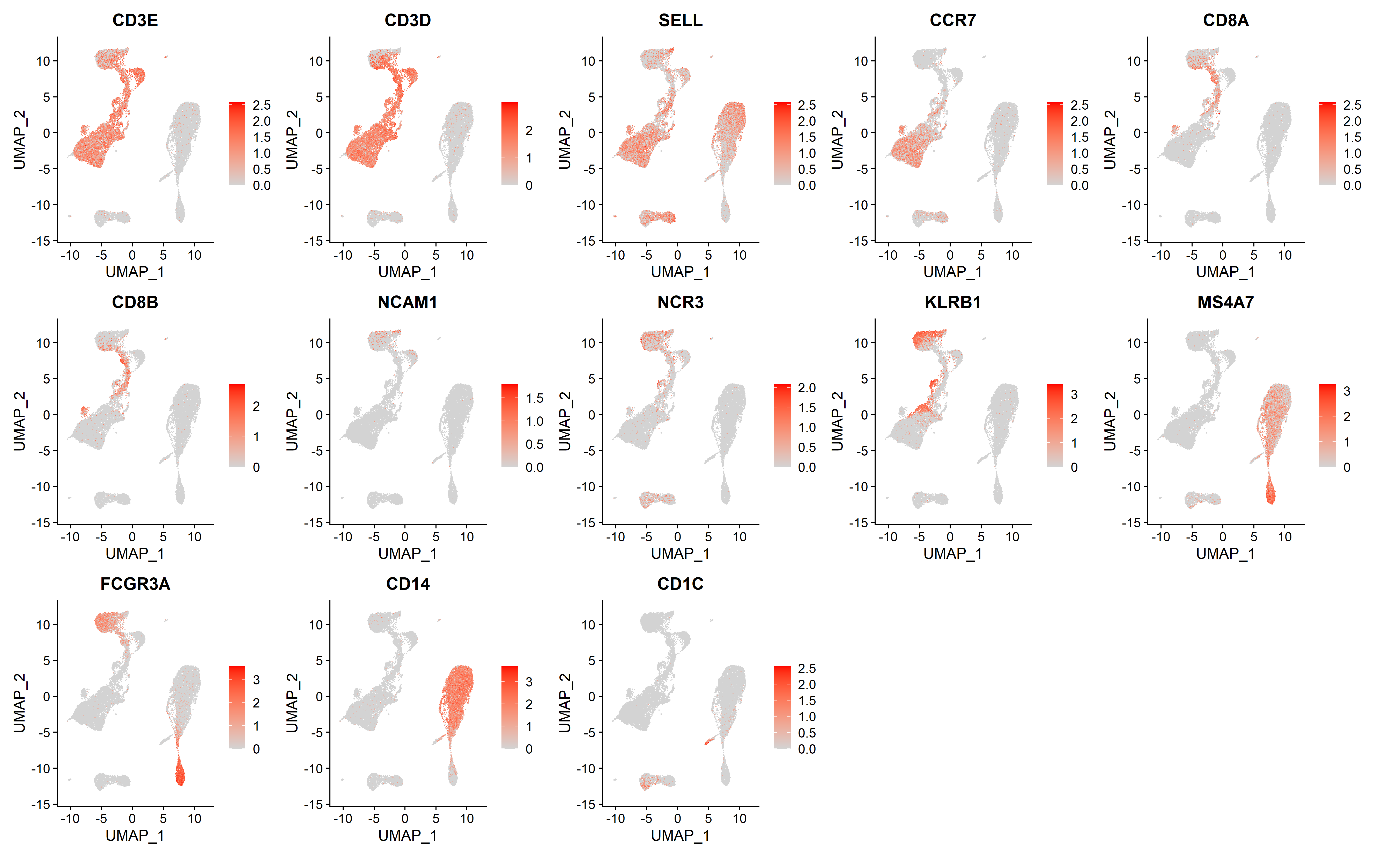
**

Feature plots of known canonical markers: naive T cells (CD3E, CD3D, SELL, CCR7), CD8+ effector T cells (CD3E, CD3D, CD8A, CD8B), CD56-high NK cells (NCAM1^high^, NCR3, KLRB1), CD16-high NK cells (FCGR3A^high^, NCR3, KLRB1), monocytes (MS4A7), classical monocytes (CD14^+^FCGR3A^−^), intermediate monocytes (CD14^+^ FCGR3A ^+^), nonclassical monocytes (CD14^dim^ FCGR3A^+^), and mDCs (CD1C).

**Figure S9. Macrophage and mDC annotation for PD gingiva (GSE164241)**


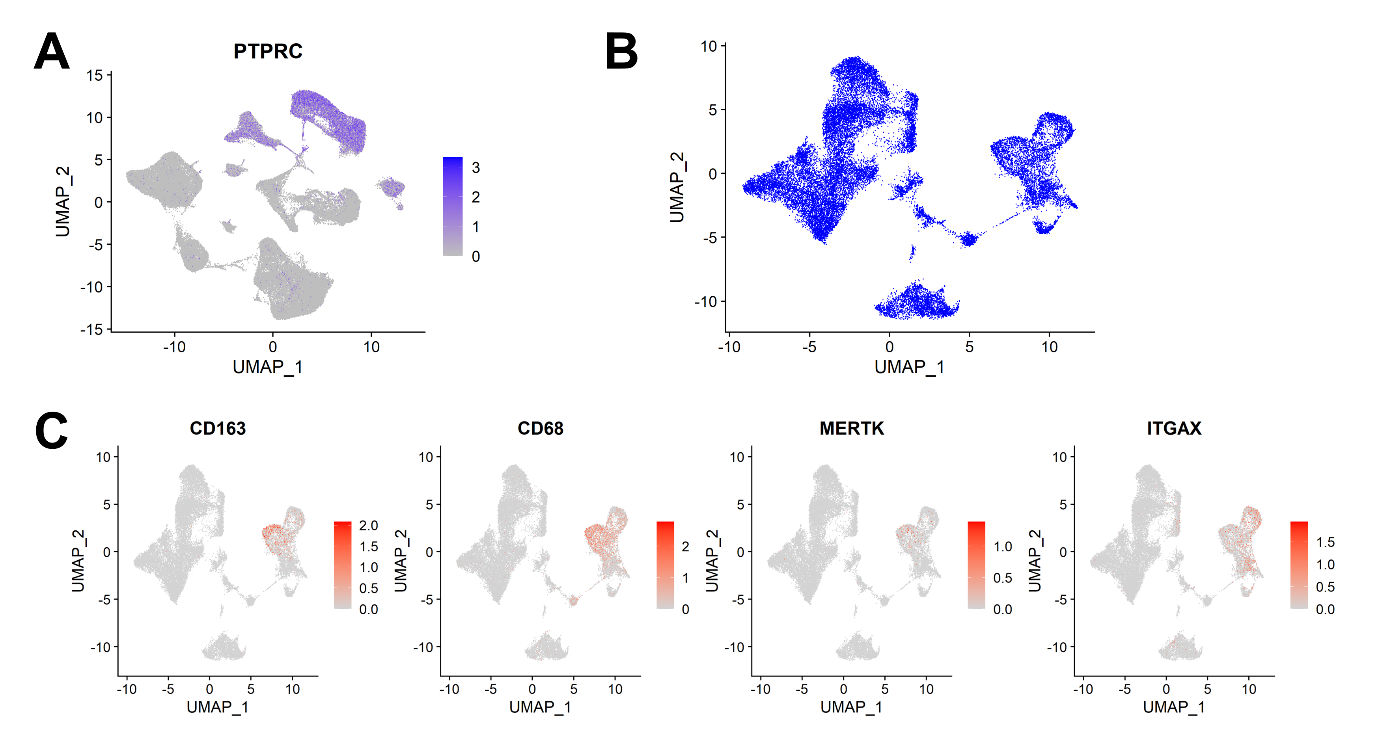


1. PTPRC expression in whole gingival cells for leukocyte selection
2. UMAP of gingival immune cells
3. Identification of macrophages and mDCs: Macrophages (CD163, CD68, and MERTK) and mDCs (ITGAX).

**Funding Information**

This work was supported by the Medical Research Center program [NRF-2018R1A5A2023879] and the Basic Science Research Program [RS-2023-00207946] through the National Research Foundation of Korea and the Korea Health Technology R&D Project through the Korea Health Industry Development Institute (KHIDI) (HI22C1377) funded by the Ministry of Health & Welfare, Republic of Korea. This study was supported by KREONET.

1. Medical Research Institute, Pusan National University, Yangsan, Republic of Korea, Yangsan, Republic of Korea, 50612 [↑](#footnote-ref-1)
2. Department of Periodontology, School of Dentistry, Pusan National University, Yangsan, Republic of Korea, 50612 [↑](#footnote-ref-2)
3. Department of Oral and Maxillofacial Surgery, School of Dentistry, Pusan National University, Yangsan, Republic of Korea, 50612 [↑](#footnote-ref-3)
4. Department of Periodontology, Dental and Life Science Institute, School of Dentistry, Pusan National University, Yangsan, Republic of Korea, 50612 [↑](#footnote-ref-4)
5. Department of Periodontology and Dental Research Institute, Pusan National University Dental Hospital, Yangsan, Republic of Korea, 50612 [↑](#footnote-ref-5)
6. Periodontal Disease Signaling Network Research Center, School of Dentistry, Pusan National University, Yangsan, Republic of Korea, 50612 [↑](#footnote-ref-6)
7. Department of Biomedical Informatics School of Medicine, Pusan National University, Yangsan, Republic of Korea, 50612 [↑](#footnote-ref-7)
8. Department of Anatomy, School of Medicine, Pusan National University, Yangsan, Republic of Korea, 50612 [↑](#footnote-ref-8)
9. Department of Oral Pathology, Dental and Life Science Institute, Pusan National University, Yangsan, Republic of Korea, 50612 [↑](#footnote-ref-9)
